# PARP1 as an Epigenetic Modulator: Implications for the Regulation of Host-Viral Dynamics

**DOI:** 10.3390/pathogens13020131

**Published:** 2024-01-30

**Authors:** Asher A. Sobotka, Italo Tempera

**Affiliations:** 1 Wistar Institute, Philadelphia, PA 19104, USA; 2 Department of Biology, University of Pennsylvania, Philadelphia, PA 19104, USA

**Keywords:** PARP1, DNA virus, epigenetic, viral gene regulation

## Abstract

The principal understanding of the Poly(ADP-ribose) polymerase (PARP) regulation of genomes has been focused on its role in DNA repair; however, in the past few years, an additional role for PARPs and PARylation has emerged in regulating viral-host interactions. In particular, in the context of DNA virus infection, PARP1-mediated mechanisms of gene regulations, such as the involvement with cellular protein complexes responsible for the folding of the genome into the nucleus, the formation of chromatin loops connecting distant regulatory genomic regions, and other methods of transcriptional regulation, provide additional ways through which PARPs can modulate the function of both the host and the viral genomes during viral infection. In addition, potential viral amplification of the activity of PARPs on the host genome can contribute to the pathogenic effect of viral infection, such as viral-driven oncogenesis, opening the possibility that PARP inhibition may represent a potential therapeutic approach to target viral infection. This review will focus on the role of PARPs, particularly PARP1, in regulating the infection of DNA viruses.

## 1. PARP Overview

Poly (ADP-ribose) polymerase (PARP) proteins are a family of proteins responsible for the transfer of single or multiple ADP-ribose moieties to protein acceptors utilizing the NAD+ as substrate, a process labeled Mono-ADP-ribosylation (MARylation) or Poly-ADP-ribosylation (PARylation), respectively [1,2,3]. The addition of these long moieties to target proteins, especially by PARP1 and PARP-2, has been primarily associated with several regulatory mechanisms, including DNA repair and programmed cell death [4,5,6,7]. However, the discovery of an increasing number of novel proteins capable of PARylation has been accompanied by an increase in known targets of PAR modifications [7]. The ability of PARylation to directly modify the structure and activity of proteins and associated elements in addition to functioning as a recruiter for additional proteins indicates that PARylation is capable of multiple means of regulation [7].

Early work regarding PARPs (especially PARP1) has focused on the DNA repair mechanisms of PARPs and PARP1-induced cell death [1,4,8]. This mechanism relies on charged ADP-ribose moiety polymers recruiting proteins responsible for DNA repair [9]. PARP1-dependent cell death—parthanatos—is enabled by high levels of PARP1 activity resulting in DNA fragmentation along with depletion of cellular NAD+ and ATP [10,11]. In addition to DNA repair mechanisms, the presence of PAR can recruit proteins responsible for other regulatory pathways [12]. While seventeen unique PARPs have been discovered, only five (PARP1, PARP-2, PARP-3, tankyrase-1, and tankyrase-2) have been directly associated with PARylation, while the others have not been shown to build ADP-ribose polymers [13,14,15]. Additionally, in regard to DNA damage, PARP1 is responsible for the majority of PARylation activity, while the other PARPs have more limited—and potentially more specific—regulatory roles [14]. While less work has been conducted regarding Mono-ADP-Ribosylation, there is evidence of similar roles of MARylation in cellular processes [16].

## 2. The Role of PARP1 in the Regulation of the Epigenome

PARylation is commonly associated with DNA repair. However, several epigenetic regulatory pathways utilize PARP1 and PARylation to modify chromatin structure and regulate gene expression. It is beyond the scope of this review to provide an exhaustive and comprehensive overview of the role of PARP1 on transcription, for which we refer readers to the works of Kraus, Ko and Ren, and Huang and Kraus [17,18,19]. Nevertheless, essential epigenetic mechanisms regulated by PARP1 and are eventually relevant to virus infection are further discussed.

### 2.1. PARPs’ Regulation of Nucleosome Structure

PARP1 and PARylation regulate transcription through multiple mechanisms that directly interact with and remodel chromatin structure, due to the ability of PARP1 to bind to DNA [17]. PARP1 can create compact chromatin structures comparable in function to H1 repressed chromatin structures [17,20]. Specifically, the DNA-binding domain (DBD) containing PARP1’s Zinc regions is involved individually in transcriptional repression and works cooperatively with the catalytic domain (CAT) to form condensed chromatin structures [20]. This N-terminal DNA binding domain consists of two Zinc fingers responsible for DNA interaction. PARP1’s zinc finger 2 has a strong affinity for DNA breaks (enabling PARP1’s DNA repair mechanism), while zinc finger 1 is responsible for the activation of PARylation activity [9]. The DBD, in conjunction with the catalytic domain of PARP1, can both condense individual nucleosome regions and form condensed structures of adjacent nucleosomes [20]. The DBD is crucial for the recruitment of PARP1 to chromatin, enabling the CAT activity on chromatin by “tethering” PARP1 to the DNA, which in turn can condense individual nucleosomes [20]. Furthermore, the CAT was found to bring together multiple nucleosomes into a denser structure through the catalytic activity that promotes PARP1 dimerization, drawing together multiple PARP1-bound nucleosomes, which creates a further repressed chromatin structure [20]. PARP1 is also involved in inhibiting this process, as PARP1 loses its nucleosome affinity when it is PARylated at the BRCT region, located in the middle core domain of the protein, which is usually referred to as the auto-modification domain [21,22,23,24,25]. PARP1 auto-PARylation results in the loss of the chromatin-compacted structures [17,20]. Together, these functions provide a picture of the specific roles of the domains of PARP1 in condensing chromatin structure: the DBD recruits PARP to the chromatin structure, whereas the CAT provides the enzymatic activity required for creating the structure, and the BRCT provides a spot for the auto modification responsible for reversing this process. This role of BRCT implies that the control of PARP enzymatic function determines both the condensing and opening of chromatin through nucleosome PARylation and auto-PARylation, respectively.

In addition to the cooperation of the DBD and CAT domains to repress chromatin, the DBD of PARP1 is capable of inhibiting transcription through the repression of RNA Pol II activity when bound to chromatin, meaning that when attached to chromatin, PARP1 has multiple methods to regulate transcription [20]. This method of transcriptional regulation is independent of the previously described nucleosome compaction but functions simultaneously, providing a cooperative method of transcriptional repression [20].

### 2.2. The Regulation of CTCF by PARP1

**Figure 1:**
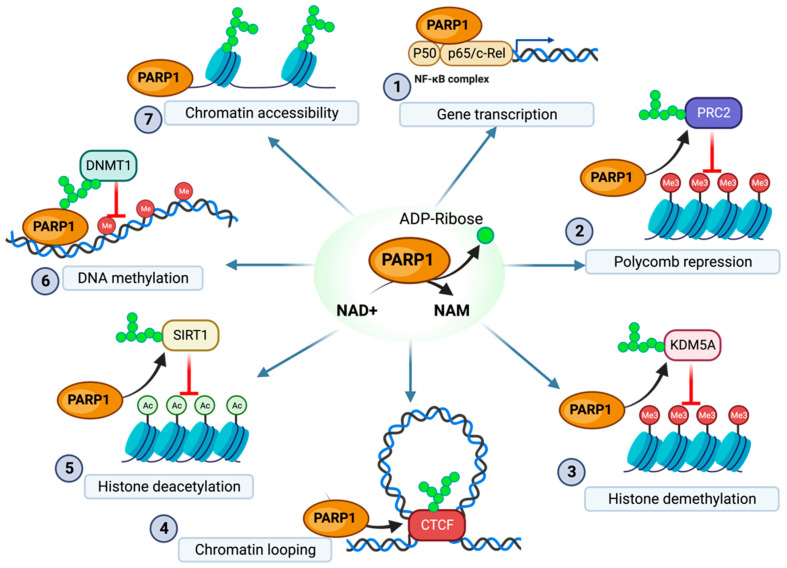
Overview of PARP1 regulation of epigenetic modification. (1) PARP1 binding to the NF-κB complex enables gene transcription of immune responses (2) PARP1 PARylation of the EZH2 component of the PRC2 complex downregulates H3K27 methylation. (3) PARylation of KDM5A inhibits histone demethylation. (4) PARylation of CTCF enables stable formation of chromatin loops (5) PARylation of SIRT1 decreases SIRT1 histone deacetylation activity. (6) PARylation of DNMT1 decreases DMNT1 methyltransferase activity. (7) Direct PARylation of histones prevents the formation of dense inaccessible chromatin structure. NAM, nicotinamide. The figure was created with Biorender.

The CCCTC-binding factor (CTCF)—a highly conserved zinc-finger protein—has been implicated in transcriptional regulation by remodeling the three-dimensional structure of the chromatin [26]. CTCF has been identified in the formation of chromatin loops associated with transcriptional activation, insulation, and silencing [26,27]. Additionally, CTCF can function as an activator of PARP1 and is regulated in part via PARylation [17,28,29,30,31] (Figure 1). CTCF recruits PARP1—in the absence of the usual stress signals such as DNA damage—both to stabilize its own binding to chromatin and to recruit PARP1 for chromatin modifications [17,31]. While PARP1 recruited by CTCF can have multiple targets (such as CTCF itself), a common target of PARylation by CTCF-recruited PARP1 is PARP1 itself, suggesting that CTCF can function as a regulator of auto-PARylation [17,31]. Additionally, PARG has been identified as reversing the modifications made by CTCF-recruited PARP1, suggesting that these modifications are transient in many cases [29]. The high levels of PARG and associated PAR depletion have even been shown to remove CTCF binding from key regulatory locations [32].

### 2.3. The Regulation of Histone Modifications by PARP1

PARP1 is associated with the repression of transcription through direct interactions with chromatin structure; conversely, PARP1 has been associated with the upregulation of transcription through histone interactions and modifications (Figure 1). PARP1 promotes transcription through the PARylation of histone H1 and core histones, which results in increased transcription [33]. This PARylation resulted in the opening of condensed chromatin structures due to the negative charge of the PAR polymer, weakening the chromatin–histone interaction and allowing for increased transcription. Although the primary histone acceptor of PARylation is H1, additional core histones are essential targets of PARylation, especially in H1-depleted chromatin structures [33]. For example, PARP1 activity has been associated with chromatin opening due to the PARylation of chromatin-bound histone H2B, which inhibits normal histone interaction with the DNA, similarly to its effect on H1 [34,35]. These PARP1 modifications have specifically been suggested to target the amino-terminal of histone tails, indicating that potentially histone acetylation at this location would have an inhibitory effect on PARylation at this site and associated upregulation in transcription [36].

Although PARylated histones are still found to be associated with chromatin, they are incapable of forming either the H1-chromatin structures typically found in H1 condensed chromatin or other canonical H1 interactions found in condensed chromatin. PARylation addition to the H1 chromatin binding sites was found to competitively bind to linker histones, displacing these linker histones and leading to increased chromatin opening [36]. In addition to the regulation of nuclear histones, cytoplasm-localized H2B, H2A, H3, and H4 were all established as targets of PARylation at specific lysine residues localized near histone tails [36]. Furthermore, the exclusion of H1 from chromatin structures due to PARylation has been demonstrated to increase transcription due to the increasing accessibility of the transcription start promoter site [37].

In addition to directly modifying histones, PARP1 has been associated with the regulation of histone modifications, such as the downregulation of the repressive chromatin marker H3K27me3. PARP1 inhibition was associated with the increased activity of Polycomb Repressive Complex 2 (PRC2)—a protein complex responsible for the methyltransferase activity of lysine 27 on H3—resulting in decreased condensed chromatin [38]. Additionally, in BRCA2 proficient cancers, this PARP1-PRC2 interaction has been shown to inhibit NF-kB immune activity [39,40,41]. This data would suggest that PARP1 supports transcriptionally activated chromatin through the downregulation of PRC2 activity (Figure 1). This inhibition of PRC2 was determined to be a consequence of the PARylation of the EZH2 enzymatic component of PRC2, which prevents the methyltransferase activity of PRC2 due to decreasing the strength of EZH2’s interaction with histone H3 [41]. This PARylation of EZH2 results in EZH2 disassociation from the PRC2 complex in addition to the downregulation of EZH2 [42]. An additional pathway through which PARP1 modifies H3 methylation is through the PARylation of the demethylase protein KDM5B (Figure 1) [37]. PARP1 was found to preserve transcriptionally activating H3K4 trimethylation through the inhibition of KDM5B, which is responsible for H3K4me3 demethylation [37]. PARP1 was established as a critical element in preventing the KDM5B repression of target genes through H3K4me3 demethylation, as PARylated KDM5B is incapable of binding target histones [37]. Corroborating data have shown that the PARP1 PARylation of EZH2 results in EZH2 disassociation from the PRC2 complex, and subsequent downregulation of H3K27me3 [42].

Beyond the modification of histone methylation, PARP1 plays an additional secondary role in histone modification through the regulation of proteins responsible for histone acetylation. Histone H3 and H4 were found to have higher levels of acetylation in the presence of PARP (despite, of course, PARP1 having no HAT activity) [43]. PARP1, independent of activation by DNA breaks, participates in the ERK/MAP kinase signaling cascade, heightening the ERK-mediated histone H3 and H4 acetylation activity [43]. This is enabled by a pERK2-PARP1 complex. When this complex is formed, PARylated ERK2 increases the phosphorylation of ELK1, which in turn is responsible for the activation of HATs such as CBP/p300 [44]. Additionally, the ERK2-PARP1 complex is responsible for the increased PARylation of histone H1 [44]. The PARG-mediated downregulation of this activity is associated with the removal of H3 and H4 acetylation, further suggesting that the presence of PAR moieties serves to regulate H3 and H4 acetylation [45]. This allows for PARP1 to act as a promotor of H3 and H4 acetylation, where in turn PARG acts to regulate this PARP1-mediated acetylation [45].

### 2.4. The Regulation of DNA Methylation by PARP1

PARP-mediated PARylation has been associated with the regulation of DNA methylation (Figure 1). It has been identified that active PARP1 is responsible for regulating DNA methylation by interacting with the DNA-methyltransferase DNMT1 [31,45,46]. This process is regulated through auto-PARylation, which determines the extent of PARP1 activity on such protein [9]. While PARP1 itself has no direct involvement with DNA methylation, it has been identified as a regulator of DNMT1 activity [31,45,46]. Based on the proposed model, it is suggested that when PARP1 is auto-modified (PARylated) or when PARs are present, they cause DNMT1 to become catalytically inactive and, therefore, less effective at carrying out DNA methylation. [31,45,46]. These changes have been implicated in the formation of less-dense chromatin structure, presumably a cooperative process with PARP1-mediated histone H1 modifications as DNA methylation’s transcriptional inhibition has relied upon the presence of linker histones, potentially suggesting a synergistic effect [47,48]. A proposed mechanism for the PARP1-mediated regulation of methylation suggests also that PARP1, activated by the CTCF binding factor, could deactivate DNMT1 activity through the PARylation of two DNA binding domains [46,49,50]. Furthermore, CTCF binding is strengthened in a complex with DNMT1 and auto-PARylated PARP1 [2]. In addition to PARylation directly inhibiting DNMT1 activity, EZH2 (a component of the PRC2 complex) has been implicated as a “recruitment platform” for DNMT1 [28]. These data, taken with PARP1’s role in inhibiting EZH2 activity, could suggest that PARP1 is able to further regulate DNA methylation through EZH2.

### 2.5. The Regulation of Protein Acetylation by PARP

In addition to the role of PARP1 in modifying histones through acetylation pathways, there is evidence of PARP1 having a role in protein acetylation beyond histones (Figure 1) [51,52,53]. In mouse models, PARP inhibition was associated with increased Sirtuin activity, suggesting that PARP1 and PARP-2 are responsible for maintaining protein acetylation [53]. The acetylation of High-Mobility Group Box-1 (HMGB1), a mediator in the inflammatory response, was found to be upregulated through multiple PARP1-mediated mechanisms, which is critical for the transport of HMGB1 to the cytoplasm and eventual release [52]. PARP1 was found to play a role in both the activation of acetyltransferases and the deactivation of deacetyltransferases [53]. Additionally, the PARylation of HMGB1 was found to facilitate acetylation [53].

## 3. Viral Utilization of PARylation

### 3.1. Immune Response

Studies over the past two decades have provided important insights into the role that PARPs and PARylation play in regulating the immune response of host cells to pathogens. PARP1 has been identified as a mediator in the activation of host antiviral immune responses. Nuclear factor-kappa B (NF-κB) is a crucial regulator of several antiviral immune responses, and PARP1 has been shown to have an interaction with both NF-κB subunits p50 and p65, in addition to interacting with p300—a coactivator of NF-κB [53] (Figure 1). In the absence of PARP1, NF-κB-dependent immune response proteins were found not to be expressed [54,55]. NF-κB is a central player in immune and inflammatory signaling responses and is responsible for various immune responses. While most studies have focused on the role of NF-κB as a transcription factor necessary for the development of B cells, NF-κB is also critical for the development and function of T cell thymocytes, dendritic cells, macrophages, and fibroblasts [56,57,58]. Additionally, NF-kB is responsible for antiviral inflammatory activation [59,60]. NF-kB can regulate these responses partly through its activity as a regulator of transcription; NF-kB has been shown to selectively activate and deactivate transcription through its direct interaction with HAT and HDAC enzymes which regulate H4 acetylation on histones near regions responsible for immune response genes [58,61]. PARP1—through the regulation of NF-kB—could regulate several parts of both the adaptive and innate immune systems by altering chromatin structure. These data suggest that PARP1, among other proteins, acts as an activator of the cellular immune response as a stress response to viral infection. NF-kB dysregulation has also been implicated in numerous pathological processes such as tumorigenesis and progression, multiple sclerosis, and inflammatory diseases such as arthritis [62,63]. In addition to the NF-kB-related inflammatory response, PARP has been implicated in regulating inflammation through HMGB1 [64,65,66,67]. Similarly to NF-kB, HMGB1 has been identified as a central protein in activating several innate immune responses related to dendritic cells, macrophages, and programmed cell death [53]. Similarly to NF-kB, HMGB1 dysfunction has also been implicated in malignancies [68,69,70]. Several PARPs have been further implicated in signaling pathways, such as the pathways related to IFN-1 production and JAK-STAT signaling [71].

Even beyond the activation of the immune response through the PARylation of host proteins related to immune pathways, PARP has antiviral activity through its PARylation of viral proteins responsible for viral maintenance, such as the Epstein–Barr nuclear antigen 1 (EBNA1) in Epstein–Barr virus (EBV), LANA1 in KSHV, nonstructural proteins in Zika and Chikungunya (CHIKV) virus, and the nucleocapsid protein in coronaviruses [72]. Some of these modifications have been identified as inhibiting critical viral functions, suggesting that PARylation may function as an immune response [72]. However, it is beyond the scope of this review to provide a comprehensive overview of the role of PARylation as an antiviral part of the innate immune system. For a more comprehensive overview of PARylation as an antiviral function, we suggest the work of Du et al. [72].

### 3.2. DNA Virus’s Utilization of PARP

Despite PARP1 having an established antiviral role, several viruses have been implicated in utilizing PARP1 to evade immune detection and assist the virus in host-pathogen conflicts. The viral utilization of PARP1 to modify viral episomes or host genes has been implicated in helping long-term viral infection in several DNA viruses as viruses utilize PARylation to affect pro-viral changes to themselves or the host.

#### 3.2.1. Herpes Simplex Virus Type 1

Herpes simplex virus type 1, or HSV-1, is characterized by chronic long-term infection of the peripheral nervous system, enabled by HSV-1 transportation through axons to neuronal ganglia, where the long-term infection is established through latent HSV-1 infection characterized by the expression of a limited set of viral genes to evade immune detection [72,73,74]. However, despite latent infection accounting for such a large portion of HSV-1 infection, HSV-1 lytic reactivation can occur due to various stress stimuli [75].

**Figure 2:**
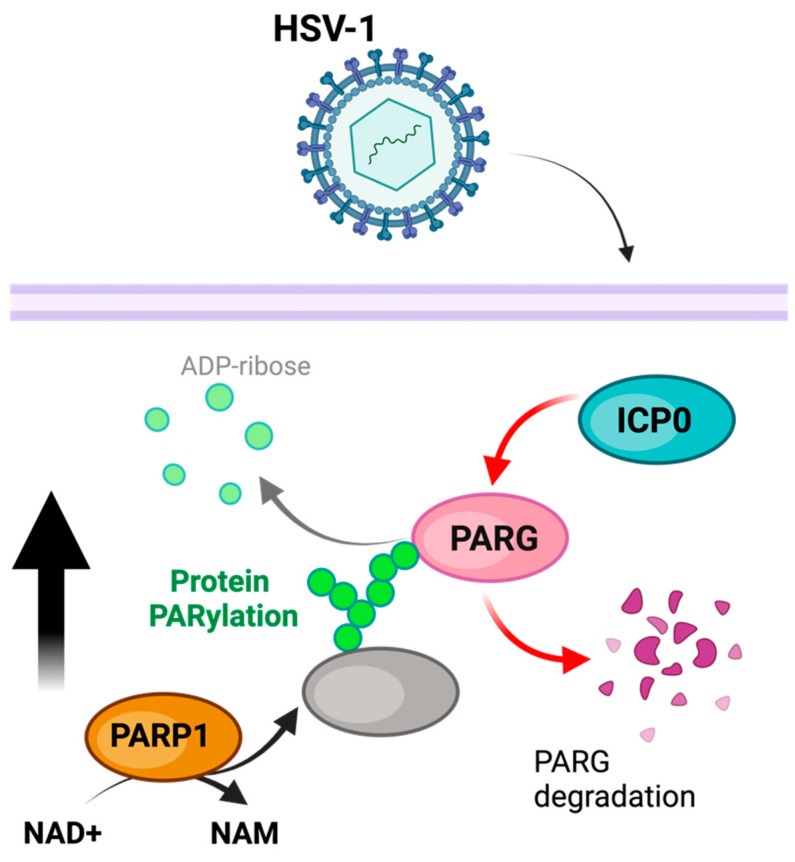
Model of PARylation during HSV-1 infection. PARP1 activity is increased, while concurrentlythe HSV-1 ICP0 degrades PARG, thereby preventing the removal of PARylation. The figure was created using Biorender.

HSV-1 infection is associated with a substantial decrease in cellular NAD+ content and an increase in PARylation [76] (Figure 2). This increase in PARylation can be reversed with PARP1 and PARP-2 inhibition, suggesting that PARP activity is responsible for these changes [77]. Furthermore, in addition to changes in PARP activity, PARG is degraded during HSV-1 infection, further implying the role of PARylation in promoting viral infection [77]. These modifications may induce the auto-PARylation of PARP1 to prevent the PARP1-mediated activation of apoptosis or parthanatos (as a stress immune response), suggesting a viral hijacking of this regulatory mechanism [77]. In addition to boosting PARP1 activity, HSV-1 has been observed to use the E3 ubiquitin ligase ICP0 to break down PARG. This process helps elevate PAR levels [78,79]. In order to prevent PARP1/2-induced cell death as part of the immune response, HSV-1 increases NAD+ levels available to PARP1/2 while degrading PARG in order to increase and maintain auto-PARylation, inhibiting PARP1/2’s stimulation of cell death. In addition to boosting PARP1 activity, HSV-1 has been observed to use the E3 ubiquitin ligase ICP0 to break down PARG. This process helps elevate PAR levels [78,79]. The aim is to safeguard against PARP1/2-triggered cell death in the immune response.

#### 3.2.2. Kaposi’s Sarcoma-Associated Herpesvirus

Kaposi’s sarcoma-associated herpesvirus (KSHV) is a γ-herpesvirus associated with long-term infection and linked to several related malignancies [28,46,80]. Long-term KSHV infection is enabled by the latent infection expression of only a limited number of viral genes [80,81].

**Figure 3:**
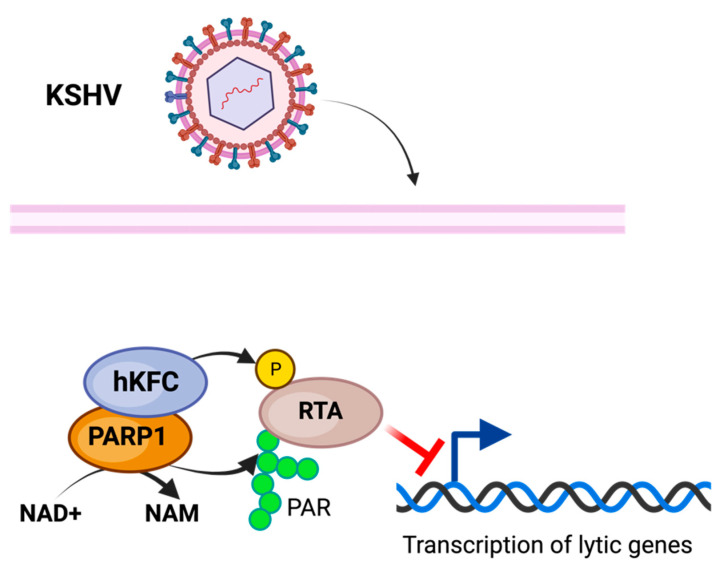
Model of PARylation during KSHV infection. Modification of KSHV transcriptional and replicative activator RTA through PARP1 PARylation along with SLK phosphorylation inhibits KSHV lytic activity, enabling latent infection. The figure was created using Biorender.

PARP1 has been associated with the repression of KSHV replication and viral expression [82]. PARP1, in conjunction with the Ste-20-like kinase hKFC, PARylate, and phosphorylate, is the KSHV replication and transcription activator (RTA), inhibiting its activity and preventing KSHV replication and transcription [83] (Figure 3). The PARP-hKFC complex enables the latent expression of KSHV by preventing the expression of genes activated through RTA [83]. The formation of the PARP1-hKFC complex is regulated through the KSHV viral processivity factor PF-8, which is responsible for the degradation of PARP1, resulting in lytic reactivation [84].

In addition to regulating KSHV latent infection, PARP1 is responsible for enabling the replication of KSHV during latent infection [84]. PARP1 has been shown to bind to KSHV’s terminal repeat sequence (which, during latent infection, functions as an origin of replication) and PARylate the latency-associated nuclear antigen associated with the terminal repeat sequence during latent infection [85].

#### 3.2.3. Epstein–Barr Virus

Epstein–Barr Virus (EBV) is a γ-herpesvirus that establishes a persistent life-long latent infection in the host cell [80,85]. However, unlike KSHV, EBV exhibits three types of latency defined by the expression of a limited number of viral genes (latent genes) in different patterns [86].

**Figure 4:**
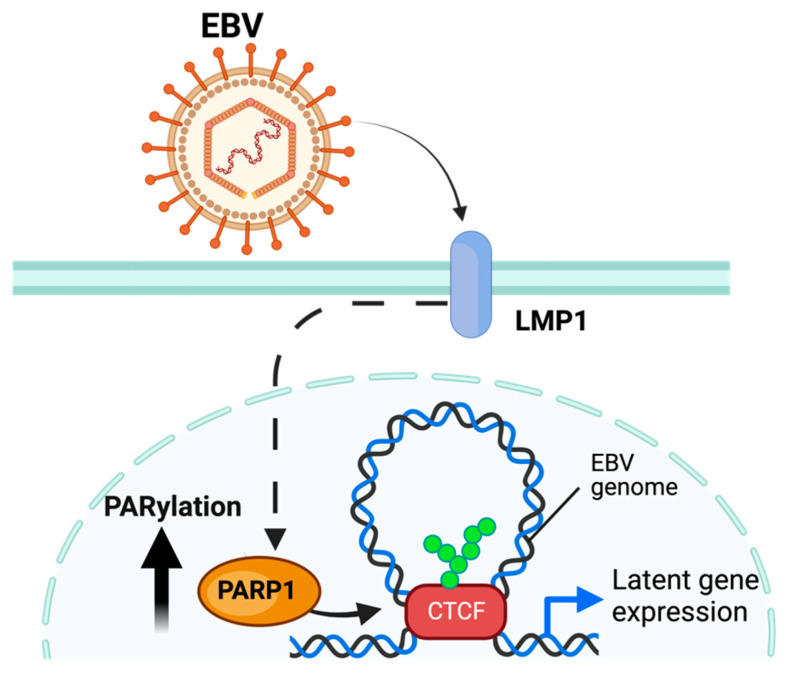
Model of PARylation during EBV infection. EBV, through the EBV LMP1 protein, increases PARP1 activity and PARylation of CTCF, enabling loop formation and expression of latent gene programs. The figure was created using Biorender.

Multiple PARPs have been ascribed a regulatory role related to EBV replication. PARP1 has been shown to directly bind to the dyad symmetry element of the EBV origin of plasmid replication (OriP), downregulating the expression of several EBV-associated genes [86,87,88] Moreover, telomere-associated PARPs (Tankerases) were also related to OriP regulation. Tankerases have been shown to bind to the dyad-symmetry elements and family of repeats region of the EBV OriP locus, a region that serves as the origin of replication of the viral genome during latent infection [89]. In addition to binding the OriP, PARP1 can modify EBV latency by binding with an additional host protein—CTCF—to the viral promoter region for the *BZLF1* viral gene, which codes the Zta protein that activates the expression of viral proteins responsible for the viral lytic replication [90,91]. This binding enables PARP1 to regulate the 3D remodeling of the EBV genome through its colocalization with other proteins, including CTCF [30] (Figure 4). The PARylation of CTCF enables loop formation to occur across the EBV genome, permitting the expression of specific viral gene programs [88,92]. In addition to maintaining latent infection, PARP1 activity has been implicated in regulating the lytic EBV cycle through similar methods [32]. This is enabled by the mechanism elucidated in Section 3.2.

In addition to PARPs modifying the EBV viral genome, PARP1 can be recruited by EBV to modify elements of the host genome. The EBV latent membrane protein 1 (LMP1) can activate hypoxia-inducible factor 1-alpha (HIF-1α) through the LMP1 activation of PARP1 mediated HIF-1α PARylation [93]. This LMP1-mediated activation of genes through PARP1 is not restricted to HIF-1α; as in the absence of competitive PARylation, increased levels of repressive histone marker H3k27me3 were found on several host genes regulated by LMP1 [94]. In addition to LMP1, EZH2, an enzymatic component of the Polycomb Repressive Complex 2 (PRC2) responsible for methyltransferase activity on H3 lysine 27, is downregulated by PARP1 PARylation (PARP1 upregulates host gene expression by decreasing global H3K27me3 through the mechanism explained above) [95]. EZH2 has also been identified as a regulator of EBV latency [38].

Besides PARP1 role in regulating the viral epigenome, EBV-transformed B-lymphocytes depend on PARP1 DNA repair functions to survive. The immune transcriptional regulator STAT3—activated during the transition from lytic EBV infection to latent infection—compromises homologous DNA repair through the inhibition of ART phosphorylation of Chk1 [96]. This results in DNA repair relying upon PARP1 contingent microhomology-mediated end-joining to the maintenance of genome integrity [96]. It is important to note that other proteins implicated in DNA mismatch repair such as PCNA are also implicated in the maintenance of EBV latency through modulating the transition from replication to transcription [97]; however, these concurrent mechanisms are beyond the scope of this review.

These data suggest that PARylation—especially PARP1-catalyzed PARylation—is responsible for both sides of the crosstalk between the EBV and the host genome. Not only does PARP1 regulate the expression of the host genome, but it is also responsible for regulating the modification of host genes relevant to EBV survival.

#### 3.2.4. Cytomegalovirus

PARP1 and PARylation also play a role in an additional herpesvirus: the human cytomegalovirus (HCMV), a β-herpesvirus [98]. Similarly to EBV’s establishment of latent infection, HCMV has been found to recruit host factors to regulate its own gene expression and enable chronic silent infection [99]. In addition to utilizing host proteins to modify viral expression, HCMV has been shown to modify cells to create a more pro-viral environment, such as by inhibiting cell death [99]. PARP1 has been linked to this regulation of HCMV infection, with HCMV infection enabling increased PARylation by PARP1, possibly due to increased DNA damage during viral infection or as a promotor of viral replication [100,101]. Further supporting the pro-viral role of PARylation in the case of HCMV, PARG has been shown to have an antiviral effect on HCMV replication [102].

#### 3.2.5. Polyomavirus

PARP1 is also implicated in the life cycle of several Polyomaviruses, including simian vacuolating virus 40 (SV40) and human polyomavirus 2 (JC virus). PARP1 is activated by and directly binds to SV40 capsid proteins [102]. PARP1 has been shown to be activated by SV40 protein VP3, inducing apoptosis which in turn enables SV40 to escape from the cell membrane [103]. However, while PARP plays a pro-viral role in SV40 replication, it has also been linked with antiviral mechanisms in response to other polyomaviruses. PARP has been implicated in several mechanisms that inhibit the replication of SV40 DNA [103]. PARP binds to the end of viral DNA Strands, competitively inhibiting the binding and activity of exonuclease III, DNA ligase, and Polα during leading strand synthesis [104]. Consequently, the presence of PARP during SV40 replication acts to limit lytic reactivation of SV40 by preventing SV40 replication of the viral genome.

#### 3.2.6. PARylation as a Tool for RNA Viruses

Even though PARPs are primarily associated with either protein or chromatin modification, there is evidence that in addition to regulating the infection of DNA viruses, PARPs also have regulatory mechanisms relevant to RNA virus function. PARP12—while only having MARylation activity—has been linked to antiviral functions regarding several alphaviruses, such as the Venezuelan Equine Encephalitis Virus (VEEV) [105].

There is emerging evidence that viral macrodomains in RNA virus families Coronaviradae, Togoviridae, and Hepeviridae themselves have enzymatic de-ADP-ribosylation activity that can offset antiviral activity by PARPs [103]. The SARS-CoV-2 MacroD-like macrodomain (Mac1)—in addition to highly similar macrodomains found in SARS-CoV and MERS-CoV—has been identified as having mono-ADP-ribose glycohydrolase activity [106,107].

There is evidence that some RNA viruses—in order to counteract PARP1’s antiviral activity—code for viral proteins with glycohydrolase activity. The SARS-CoV-2 virus encodes the nonstructural protein 3 (Nsp3) which contains a macrodomain. This macrodomain has been found to remove ADP-ribose from proteins, counteracting the increase in PARylation that occurs in response to the interferon signaling cascade. Due to the highly conserved nature of these macrodomains (26% of the amino acids differ from the SARS-CoV-2 macrodomain compared to other coronaviruses), it has been suggested that this de-PARylating activity is common among viral macrodomains [106,108]. However, despite several macrodomains being similarly capable of PAR removal, the effects of these macrodomains on viral infection vary. The macrodomain of CHIKV—a Togavirus—was identified as promoting viral replication through the de-PARylation activity [109]. Conversely, in the Mac1 macrodomain found in several coronaviruses (the macrodomain responsible for activity related to PAR), there is no evidence that it has a similar role to the CHIKV macrodomain in viral replication [106]. The coronavirus domain instead was associated with the modification of pathogenesis [106,110].

Human immunodeficiency virus (HIV-1) has been found to be able to inhibit the PARP1 activation of the NF-κB immune pathway, a pathway that, as discussed above, is highly regulated by PARP1. The HIV-1 Vpr and glucocorticoid receptor cooperatively function to form a complex with PARP1, preventing PARP1 activation of the host immune system and PARP1 antiviral activity [111]. However, there is some evidence of a pro-viral role of PARP1 in HIV-1 infection. While there is disagreement regarding to what extent PARP1 is dispensable for HIV-1, it is clear that PARP1 plays a prominent role in enabling quick integration of HIV-1 due to its ability to repair DNA breaks left by HIV-1 enzyme integrase’s insertion into the host genome of HIV-1 DNA from HIV-1 reverse transcriptase, utilizing the previously elucidated DNA repair mechanism [112,113].

## 4. Conclusions

Summarizing the role of PARP1 and PARylation in regulating host-viral conflicts is difficult as PARylation has the aforementioned mechanisms of epigenetic regulation that can both increase and decrease gene expression. Furthermore, its interaction with even genetically similar viruses can vary greatly. While PARylation has multiple antiviral mechanisms, several viruses can inhibit these antiviral functions and recruit and utilize PARPs to make pro-viral modifications to the host or the viruses themselves. These increasing number of observations have revealed the importance of PARylation and PARPs’ activity in regulating virus–host interaction. However, the increasing number of findings has brought up critical theoretical issues related to the role of PARylation and PARP1. For example, since PARylation requires adequate levels of NAD+, either antiviral or pro-viral functions of PARP necessitate a rewire of cellular metabolism to allow proper PARP function. While the modifications made by PARPs to promote viral infection are well established, how cellular metabolism is modified to enable this activity is less understood. While extensive data demonstrate the role PARylation plays in promoting viral infection, how exactly the cellular environment is changed to allow for this modification needs to be further established.

While PARPs (especially PARP1) have been identified both as vital parts of several regulatory pathways and as directors of host-viral interactions, the role glycohydrolase activity and PARG play beyond its response to PARPs is much less examined. Most work regarding PARG relates to its role in reversing the activity of PARP1; however, recent data regarding glycohydrolase activity in the macrodomains of several viruses such as SARS-CoV-2 would suggest that further investigation into glycohydrolase activity—independent of PARP1—is warranted. Even though the importance of PAR for several pro and antiviral mechanisms is well established, very little is known about PARG’s role in mediating viral activity relative to PARP1.

A better understanding of the role of PARPs and PARylation during viral infection is fundamental; while several FDA-approved PARP inhibitors exist and are used as targeted cancer drugs, their potential efficacy in treating viral infection and wider viral-associated pathogenesis has not been tested yet.

## Data Availability

Not applicable.
